# Modeling trap dynamics in oxide-engineered heterostructure TFETs for breast cancer detection

**DOI:** 10.1038/s41598-025-34105-0

**Published:** 2025-12-30

**Authors:** Rittik Ghosh, Priyanka Saha

**Affiliations:** 1 Institute for Microelectronics, TU Wien, Vienna, Austria; 2 Department of Electronics and Communication Engineering, St. Thomas’ College of Engineering and Technology, Kolkata, India

**Keywords:** Breast cancer, TFET, Traps, Sensitivity, Reliability, Hysteresis, Degradation, Engineering, Materials science, Nanoscience and technology, Physics

## Abstract

This work presents a reliability-focused modeling study of trap-induced hysteresis in a heterostructure oxide-engineered double-gated tunnel field-effect transistor based biosensor for breast cancer detection. Our study reveals how interface traps impact threshold voltage shifts, current sensitivity, and overall sensing stability under varying sweep rates and temperatures. We explore the role of trap location by comparing two critical interfaces, InAs-Si at the source-channel tunneling junction and Si-SiO_2_ at the channel-oxide interface, revealing that these traps induce more severe hysteresis and signal degradation. A novel detection inaccuracy metric is introduced to quantify sensitivity loss due to reliability degradation, showing up to 97% inaccuracy for healthy biomarker conditions in damaged devices. This study highlights the importance of reliability-aware modeling of trap effects in TFET-based biosensors and provides simulation-based insights into how sweep rate and temperature may influence stable and reproducible sensing behavior.

## Introduction

Breast cancer is among the most prevalent malignancies in women and a major cause of cancer-related deaths, responsible for 11.6% of cases and 6.6% of mortalities^1^. Although early detection improves survival, standard methods like mammography and MRI suffer from high costs, limited access, and variable accuracy^2–6^, especially in low-resource settings where affordable diagnostics are critically needed. Field-effect transistors (FET)-based biosensors have recently gained prominence due to their label-free detection, high sensitivity, complementary metal-oxide semiconductor (CMOS) compatibility, and suitability for miniaturization and integration^7,8^. Tunnel FETs (TFET), in particular, offer ultra-low power operation and steep subthreshold slope via band-to-band tunneling (BTBT)^9,10^. The dielectric modulation (DM) technique enhances cancer detection by altering the local dielectric constant near the channel that alters the gate-channel capacitance^11,12^. Breast cancer cells such as T47D and MCF-7 exhibit higher dielectric constants ($\kappa$ up to 32) than non-tumorous healthy cells such as MCF-10A ($\kappa \approx 4.5$) due to increased water content^13^. The detectable size of biomolecules in DM-TFET biosensors is primarily determined by the cavity thickness and the electric field penetration across gate–channel. In previously reported TFET-based biosensors, nanoparticles and cancer cell lines with characteristic sizes below 10 nm have been detected effectively through dielectric modulation within the sensing cavity^14–16^.

Despite considerable progress in improving the sensitivity of DM-TFET-based biosensors^12,17,18^, one critical aspect remains; the underexplored reliability of devices. In real-world biomedical applications, especially those involving repeated usage or long-term monitoring, the consistency of the sensor’s electrical response is just as important as its sensitivity and selectivity. Unfortunately, most prior studies have neglected the time-dependent degradation effects that can compromise biosensor accuracy. One of the main sources of time-dependent degradation in FET-based biosensors is threshold voltage hysteresis, which arises from charge trapping and detrapping at the semiconductor–oxide interface^19–22^. These interface traps, often introduced during fabrication steps and energetic band misalignments, can cause significant shifts in the transfer characteristics of the device when the gate voltage is swept forward and reverse. Such shifts not only affect signal reproducibility but can also result in false positives or missed detections; scenarios that are unacceptable in clinical diagnostics^21,23,24^.

Driven by these factors, we report a III-V heterostructure oxide-engineered double-gated TFET (H-OE-DG-TFET) biosensor to assess reliable prediction of breast cancer. The Indium Arsenide source-silicon channel (InAs-Si) heterostructure improves the saturation drain current ($I_{\text {ON}}$) due to its straddling electronic band alignment since InAs and Si has electronic band-gaps of 0.354 eV^25^ and 1.12 eV respectively. We simulate the effects of interface traps and their transient charge dynamics across varying dielectric constants, sweep rates (SR), and temperatures, addressing sensing, selectivity and reliability. By embedding reliability modeling into the biosensing framework, this study highlights the stability and robustness of TFET biosensors, advancing their potential for scalable, high-fidelity diagnostics by bridging the current gap between TFET device reliability research and biomedical sensing applications^18,26–31^.

**Figure 1:**
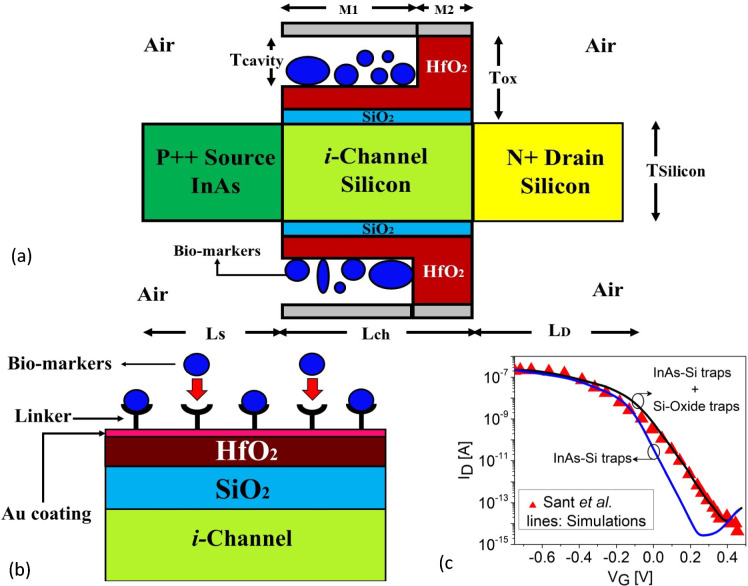
(**a**) Cross-sectional schematic of H-OE-DG-TFET biosensor device with embedded cavity. (**b**) Magnified view of the cancer cells binding to the linker molecules inside the cavities. (**c**) Calibration profile of TCAD simulations with the experimental results of *P* type InAs-Si TFET considering trap dynamics at the InAs-Si and Si-Oxide interfaces in terms of $I_{\text {D}}(V_{\text {G}})$ transfer characteristics.

## Modeling the sensor

The cross-sectional schematic of the H-OE-DG-TFET biosensor is depicted in Fig. 1a. Studies indicate a lattice mismatch $\approx$ 11% in InAs-Si heterostructures^32^. The electronic band alignment and reduced effective mass density of InAs^33^ enhance band-to-band tunneling (BTBT) by decreasing the tunneling distance ($\lambda _{\text {W}}$) at the InAs-Si junction under gate bias ($V_{\text {G}}$), thereby improving carrier mobility ($\mu$) and $I_{\text {ON}}$. To enhance SS and suppress leakage current ($I_{\text {OFF}}$), the design employs a dual material gate architecture: Aluminum ($\phi _{\text {M2}}=4.7 \, \text {eV}$) on Hafnium dioxide (HfO$_2$) and Copper ($\phi _{\text {M1}}=3.8 \, \text {eV}$) near the tunneling junction as explored in^34,35^. An oxide stack (HfO$_2$/SiO$_2$) of 2 nm thick high-$(\kappa )$/1 nm thin low-$(\kappa )$, beneath the cavity prevents additional carrier leakage, validated in^10,36^. Cavities etched in HfO$_2$ facilitate cancer cell binding (Fig. 1b), influenced by hydrophobic effects, electrostatic forces, and van der Waals interactions. Structural details are summarized in Table 1, incorporated with 30$\times$8 cavity area for cancer cell conjugation^10,37^. Simulations compare cancer cells-filled and empty cavities (air-filled, $\kappa$ =1) to evaluate the biosensor performance and long-term stability.

The H-OE-DG-TFET biosensor is simulated using the commercial SILVACO ATLAS TCAD simulator^38^. Quantum tunneling is modeled with the BBT.NONLOCAL model, using refined meshing near the InAs-Si junction with QTX.MESH, QTY.MESH. Lateral and vertical tunneling directions are specified by QTUNN.DIR=1 and 0, respectively. The CONMOB and FLDMOB models are used for concentration and electric field mobility, and the FERMI model is activated for Fermi-Dirac statistics. Carrier recombination is modeled with the Shockley-Read-Hall (SRH) and BGN models for band gap narrowing. The TRAP.TUNNEL model implements trap-assisted tunneling that accounts for a field-dependent undesirable tunneling of an electron from the valence band to the conduction band of the channel via an intermediate energy state (trap), while DRIFT DIFFUSION models handle carrier transport. Time-dependent degradation due to interface traps (switching traps) is captured by the semi-classical HEIMAN model, simulating elastic tunneling of defect charge states highlighting their impact on the $I_{\text {D}}(V_{\text {G}})$ profile during forward/reverse sweeps. The simulation framework uses $V_{\text {G}} \in$ [0 V, 1.5 V] and $V_{\text {D}} = 1 \, \text {V}$. Model validity is verified with experimental data from the *P* type InAs–Si TFET devices by Sant et al.^25^ by adjusting the interface trap density ($N_{\text {it}}$) and carrier mobility ($\mu$) for subthreshold slope (SS) and saturation drain current ($I_{\text {ON}}$) matching, as shown in Fig. 1c. The best agreement with the experimental transfer curve is obtained for $N_{\text {it}}$ of the order of $10^{12}~\text {cm}^{-2}\,$ at the silicon-silicon dioxide (Si–SiO$_2$) interface and a calibrated channel mobility of $590~\text {cm}^{2}\text {/V} \text {s}$, as shown in Fig. 1c and consistent with reported TFET values in the literature^39^. The impact of traps at both the InAs-Si and Si-Oxide interfaces were implemented in the simulation framework. Furthermore, we extended this analysis to show the hysteresis behavior over varying sweep rates to observe device degradation from both of these interfaces.

**Figure 2:**
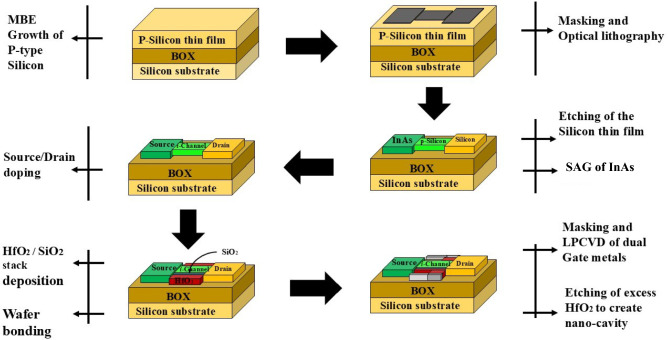
3-D possible fabrication process flow of H-OE-DG-TFET-based biosensor device.

The fabrication feasibility of the H-OE-DG-TFET biosensor is strongly supported by recent experimental works on InAs-Si integration. Moselund et al.^40^ demonstrated lateral *P* type InAs-Si TFETs monolithically integrated on silicon-on-insulator substrates using template-assisted selective epitaxy. Their process employed in-situ Si$_2$H$_6$ doping of InAs within SiO$_2$ templates and a gate stack composed of Aluminum oxide-Hafinum dioxide-Tungsten (Al$_2$O$_3$-HfO$_2$-W) closely resembling the oxide–metal configuration adopted in this work. These results validate that selective-area growth (SAG), precise InAs doping, and lateral template formation are experimentally realizable. The possible fabrication process as inspired from^40^ is outlined for H-OE-DG-TFET biosensor using a 3-D schematic shown in Fig. 2. The cavity is formed by wet-etching the oxide. As a side note, wet-etching of the cavity can result in sub-par cavity-oxide interface^12,41^. The $M_{\text {1}}$ gate metal is deposited on HfO_2_-SiO_2_-Si by Electron-Beam (E-Beam) Evaporation^42^, followed by a hard mask and lithography to define the $M_{\text {1}}$ region. After selective etching, $M_{\text {2}}$ is deposited in the etched regions, completing the dual metal gate configuration.

**Table 1:** Modeling parameters of H-OE-DG-TFET-based biosensor device^43,44^.

Parameters	Values
Silicon thickness ($T_{Si}$)	10 nm
Oxide thickness ($T_{ox}$)	11 nm
Cavity thickness ($T_{cavity}$)	8 nm
Channel length ($L_{ch}=M_1+M_2$)	30 nm+10 nm
Source length ($L_S$)	25 nm
Drain length ($L_D$)	35 nm
P++ Source doping ($N_{S}$)	$1 \times 10^{19} \, \text {cm}^{-3}$
Intrinsic channel doping ($N_{ch}$)	$1 \times 10^{15} \, \text {cm}^{-3}$
N+ Drain doping ($N_{D}$)	$5 \times 10^{18} \, \text {cm}^{-3}$
Gate metal1 Work function ($\phi _{M1}$)	3.8 eV
Gate metal2 Work function ($\phi _{M2}$)	4.7 eV

**Figure 3:**
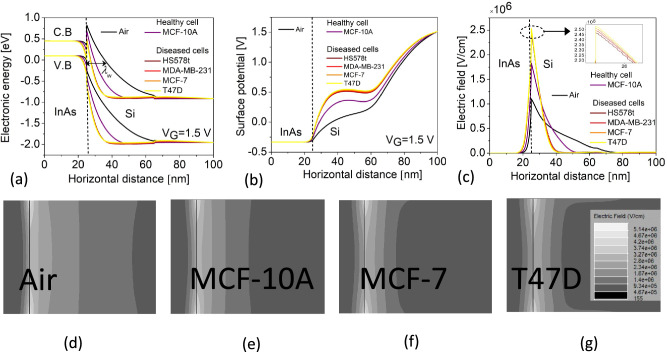
(**a**) Energy band diagram depicting BTBT (**b**) Surface potential profile (**c**) Electric field vs channel horizontal cut-line. Electric field contour profiles at the source-channel tunneling junction for (**d**) air ($\kappa$ =1) (**e**) Healthy cell, MCF-10A ($\kappa$ =4.5) (**f**) Diseased cells, MCF-7 ($\kappa$ =27.5) and (**g**) T47D ($\kappa$ =32) filled cavity.

**Figure 4:**
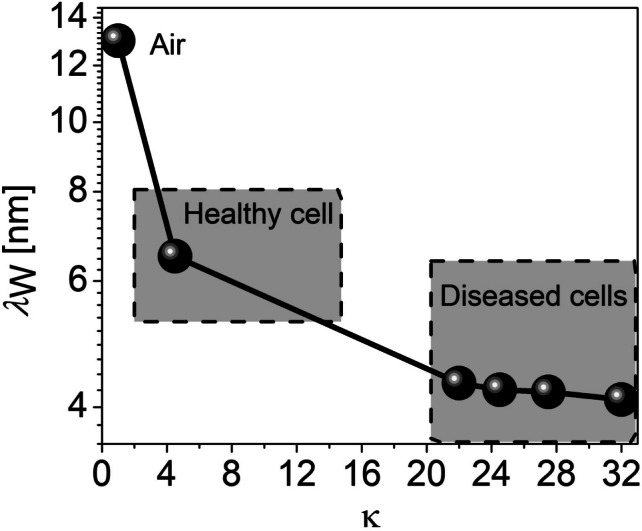
Variation of the tunneling distance ($\lambda _{\text {W}}$) as a function of the dielectric constants ($\kappa$) of the cancer cells distinguishing diseased cells to healthy cells relative to the empty cavity.

**Figure 5:**
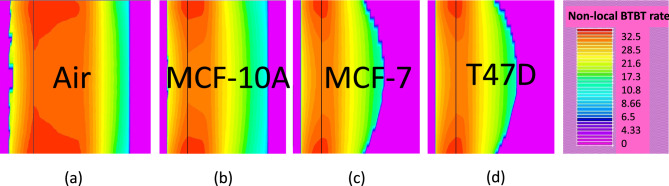
BTBT rates contour profiles for (**a**) air ($\kappa$ = 1) (**b**) Healthy cell, MCF-10A ($\kappa$ = 4.5) (**c**) Diseased cells, MCF-7 ($\kappa$ = 27.5) and (**d**) T47D ($\kappa$ =32) filled cavity.

## Simulation results

This section discusses the electrostatics and sensing performance of the H-OE-DG-TFET biosensor for breast cancer cell: (a) fresh biosensor (interface traps free) and (b) damaged biosensor (with interface traps). The performance of the sensor is shown in Fig. 3, with electrostatics extracted along the channel horizontal cut-line at maximum gate voltage ($V_G = 1.5 \, \text {V}$). The BTBT phenomenon is depicted in the energy band diagram (Fig. 3a). Carrier tunneling from the InAs valence band maxima (VBM) to the Si conduction band minima (CBM) improves as $\lambda _{\text {W}}$ shrinks with increasing $\kappa$ values of cancer cells in the cavity, compared to the empty cavity ($\kappa$ =1). Diseased cells with higher $\kappa$ result in significant band bending, increasing net oxide capacitance (C$_{\text {net}}$) and affecting the flat-band voltage ($V_{\text {FB}}$), increasing the surface potential ($\psi _s$) and perpendicular electric field at the tunneling junction (Fig. 3b–c). Contour profiles in Fig. 3d–g visually depict the strengthening electric field due to improved BTBT rates at the tunneling junction. For diseased cells, $\lambda _{\text {W}}$ narrows by approximately 70%, enhancing BTBT rates, as shown in Fig. 4 and further represented along the channel horizontal cut-line contour profiles in Fig. 5a–d. The BTBT rates are governed by the Wentzel-Kramers-Brillouin (WKB) tunneling probability as a function of electric field (E), derived from the second-order time-independent Schrödinger equation (Eq. (1)).

1 $$\begin{aligned} \log (T_E) \propto -\sqrt{m^* E_g} \lambda _{\text {W}} \end{aligned}$$

**Figure 6:**
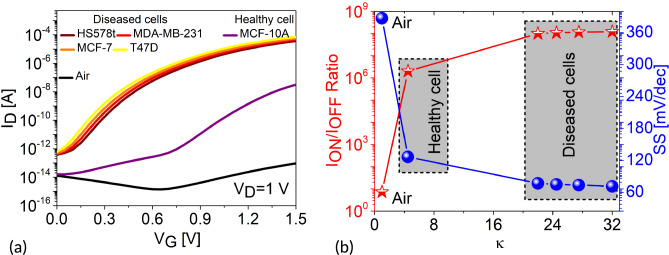
(**a**) $I_{\text {D}}(V_{\text {G}})$ profile (**b**) $I_{\text {ON}}$/$I_{\text {OFF}}$ ratio and SS vs cancer cell dielectric constants $\kappa$ values in the cavity for neutral cancer cells.

Here, $m^*$, $E_g$, and $\lambda _{\text {W}}$ represent the carrier effective mass undergoing tunneling and semiconductor electronic band gap energy respectively. This establishes the inverse relation between $T_E$ and $\lambda _{\text {W}}$ that explains the variations in electrostatics so far. Considering these, next we investigate their impact on the steady-state $I_{\text {D}}(V_{\text {G}})$ profile under neutral conditions (fixed inteface charge density ($N_{\text {c}}$) = 0) as shown in Fig. 6a. Upon sweeping the $V_{\text {G}}$ to 1.5 V, significantly higher $I_{\text {ON}}$ is extracted for diseased cells when compared to the healthy cell. This holds true based on the analysis for increasing BTBT rates for higher $\kappa$ values of the cancer cells binding to the linker molecules in the cavity. Besides, it is known from^37^ that activating TAT model (TRAP.TUNNEL) degrades the SS, making it closer to real-time values^45^. Figure 6b depicts $I_{\text {ON}}$/$I_{\text {OFF}}$ ratio and the SS as function of the cancer cell $\kappa$ values. Table 2 compacts these values. It is observed that the transistor switching improves by eight orders of magnitude while SS steepens by 81% for diseased cells.

**Figure 7:**
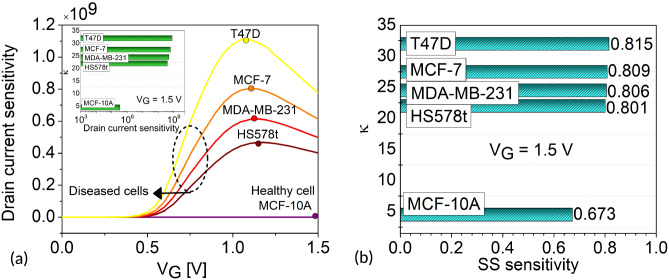
(**a**) Drain current sensitivity as a function of $V_G$. Inset shows drain current sensitivity for different cancer cells $\kappa$ values at $V_G$= 1.5 V (**b**) sub-threshold slope sensitivity for various $\kappa$ values of cancer cells at $V_G$= 1.5 V and $N_{\text {c}}$ = 0.

**Figure 8:**
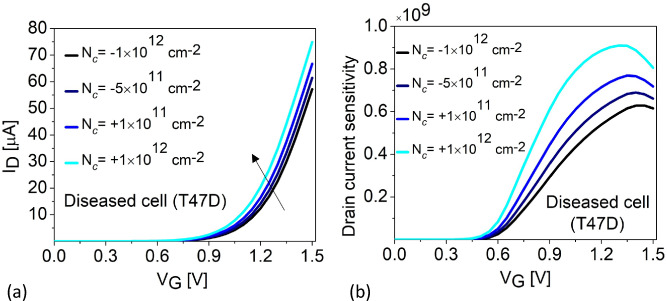
(**a**) Linear $I_{\text {D}}(V_{\text {G}})$ profile (**b**) Drain current sensitivity as a function of $V_G$ for varied T47D $N_{\text {c}}$.

**Table 2:** Impact of different cancer cells on electrostatics.

Cancer cells	I$_\text {ON}$/I$_\text {OFF}$	SS [mV/dec]
Air $(\kappa =1)$	7.2	387
MCF-10A $(\kappa =4.5)$	$2.01 \times 10^{6}$	126.5
HS578t $(\kappa =22)$	$0.994 \times 10^{8}$	77
MDA-MB-231 $(\kappa =24.5)$	$1.08 \times 10^{8}$	75
MCF-7 $(\kappa =27.5)$	$1.16 \times 10^{8}$	73.8
T47D $(\kappa =32)$	$1.2 \times 10^{8}$	71.4

### Sensitivity for a fresh biosensor

Our results confirm that $I_{\text {ON}}$ and SS are excellent candidates for sensing parameters. Sensitivities are estimated for the biosensor performance, considering cancer cell occupied cavities with respect to an empty cavity. In this work, we investigate the drain current sensitivity ($S_{I_{\text {ON}}}$) and sub-threshold slope sensitivity ($S_{SS}$) of the H-OE-DG-TFET biosensor based on^46,47^, as shown in equations (2) and (3), respectively. Figure 7a shows $S_{I_{\text {ON}}}$ as a function of the $V_G$ sweep for healthy and diseased cells at $N_{\text {c}}$ = 0. From the Gaussian-like curve, peak $S_{I_{\text {ON}}}$ of $1.11 \times 10^{9}$ is extracted for T47D diseased cells at $V_G = 1.05 \, \text {V}$, while the healthy cell, MCF-10A, yields a peak $S_{I_{\text {ON}}}$ of $3.42 \times 10^{5}$ at $V_G = 1.5 \, \text {V}$. Overall, the diseased cells yield higher peak $S_{I_{\text {ON}}}$ at lower $V_G$ values compared to the healthy cell, quantified in Table 3. This establishes $S_{I_{\text {ON}}}$ as an excellent sensing metric for breast cancer detection. For comparison, $S_{I_{\text {ON}}}$ is also calculated for a fixed $V_G = 1.5 \, \text {V}$, shown in the inset of Fig. 7a. The trend of increasing sensitivities for diseased cells compared to healthy cells is also observed for the $S_{SS}$ profile as shown in Fig. 7b. The $S_{SS}$ improves by 17.4% for T47D compared to MCF-10A. Both of these sensing metrics are quantified in Table 4. For completeness, we have evaluated the biosensing performance in the presence of charged diseased cancer cell, T47D, in Fig. 8. This is implemented using the MOSFET voltage balance equation, $V_{GS} = \psi _s + \phi _{ms} - \left( \dfrac{q(\pm N_c)}{C_{\text {eff}}} \right)$, where $N_c$ is the density (fixed) of charged cancer cells, $C_{\text {eff}}$ is the effective capacitance per unit area, and $\phi _{ms}$ is the metal–semiconductor work function. The charge density of T47D is varied from $N_{\text {c}}$ = -$1 \times 10^{12}$ $\text {cm}^{-2}$, -$5 \times 10^{11}$ $\text {cm}^{-2}$ to $N_{\text {c}}$ = +$1 \times 10^{11}$ $\text {cm}^{-2}$, +$1 \times 10^{12}$ $\text {cm}^{-2}$. Adding fixed positive charges at the cavity-oxide interface results in a strong inversion, meaning that the sensor switches ON quickly, increasing BTBT rate, thereby shifting the threshold voltage towards the lower gate voltages and simultaneously fixed negative charges makes it difficult for the sensor to switch ON due to strong accumulation at the cavity-oxide interface, meaning that the threshold voltage shifts towards the higher gate voltages as shown from the linear $I_{\text {D}}(V_{\text {G}})$ transfer characteristics in Fig. 8a. Furthermore, we calculated $S_{I_{\text {ON}}}$ for the charged T47D cancer cell for varying gate voltages in Fig. 8b where we observed peak $S_{I_{\text {ON}}}$ of $9 \times 10^{8}$ at $V_G = 1.35 \, \text {V}$ for $N_{\text {c}}$ = +$1 \times 10^{12}$ $\text {cm}^{-2}$ and $S_{I_{\text {ON}}}$ of $0.65 \times 10^{9}$ at $V_G = 1.4 \, \text {V}$ for $N_{\text {c}}$ = -$1 \times 10^{12}$ $\text {cm}^{-2}$.

2 $$\begin{aligned} S_{I_{\text {ON}}} = \frac{I_{ON}(\text {Cancer cell})}{I_{ON}(\text {Air})} \end{aligned}$$

3 $$\begin{aligned} S_{SS} = \frac{|SS(\text {Cancer cell}) - SS(\text {Air}) |}{SS(\text {Air})} \end{aligned}$$

**Table 3:** Peak drain current sensitivity at different $V_G$ values for $N_{\text {c}}$ = 0.

Cancer cells	Peak $V_G$ [V]	Peak $S_{I_{\text {ON}}}$
MCF-10A $(\kappa =4.5)$	1.50	$3.42 \times 10^{5}$
HS578t $(\kappa =22)$	1.16	$4.67 \times 10^{8}$
MDA-MB-231 $(\kappa =24.5)$	1.15	$6.14 \times 10^{8}$
MCF-7 $(\kappa =27.5)$	1.10	$8.04 \times 10^{8}$
T47D $(\kappa =32)$	1.05	$1.11 \times 10^{9}$

**Table 4:** Sensitivity at fixed $V_G$ for $N_{\text {c}}$ = 0.

Cancer cells	${\boldsymbol{S}}_{SS}$	$S_{I_{\text {ON}}}$
MCF-10A $(\kappa =4.5)$	0.673	$3.42 \times 10^{5}$
HS578t $(\kappa =22)$	0.801	$4.01 \times 10^{8}$
MDA-MB-231 $(\kappa =24.5)$	0.806	$4.98 \times 10^{8}$
MCF-7 $(\kappa =27.5)$	0.809	$6.13 \times 10^{8}$
T47D $(\kappa =32)$	0.815	$7.77 \times 10^{8}$

### Modeling hysteresis in damaged biosensors

**Figure 9:**
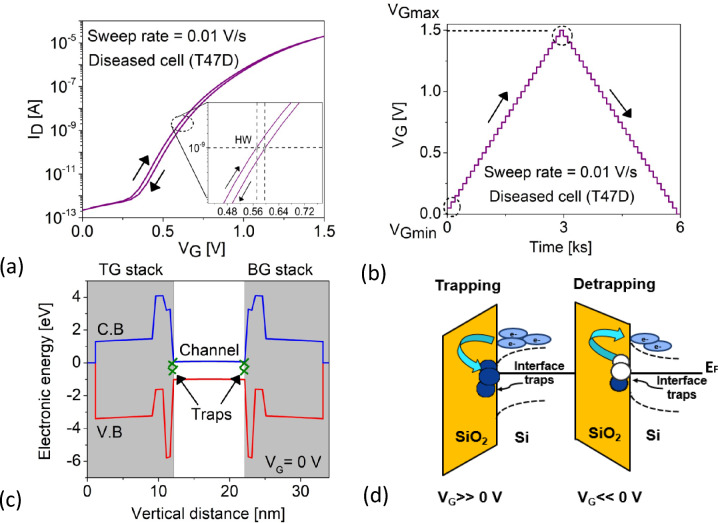
(**a**) $I_{\text {D}}(V_{\text {G}})$ profile with transient trapping showing HW (**b**) triangular waveform of the $V_G$ signal over forward/reverse sweep (**c**) energy band diagram along the gate stack highlighting the interface traps at flat-band voltage (**d**) schematic of energy bands showing electron trapping and detrapping during up and down sweeps at Si-SiO$_2$ interface and $N_{\text {c}}$ = 0.

Researchers have mainly focused on the steady-state analysis of fresh (trap-free) biosensor devices, but their reliability and stability analysis for industrial applications are equally important. Silicon forms the best interface with its native oxide, SiO$_2$, resulting in small defect density, typically P$_b$ centers^48,49^. However, to the best of our knowledge, little theoretical research has explored the impact of transient trap dynamics at the semiconductor-oxide interfaces of biosensor devices, that leads to charge trapping and detrapping during a gate bias sweep, underscoring device performance in the process. This is attributed from the threshold voltage ($V_{\text {th}}$) drifts during the forward/reverse sweep of the $I_{\text {D}}(V_{\text {G}})$, leading to hysteresis width (HW)^19,50–52^:

4 $$\begin{aligned} \text {HW} = |V_{\text {th}}(\text {for}) - V_{\text {th}}(\text {rev}) |= \frac{qf_tN_{\text {it}} }{C_{\text {net}}} \end{aligned}$$

where *q* is the elemental charge constant, and $N_{\text {it}}$ is the equivalent trap density which is a function of the trap occupation probability ($f_t$) and the net capacitance $\left( \dfrac{1}{C_{\text {net}}} = \dfrac{1}{C_{\text {ox}}} + \dfrac{1}{C_{\text {ch}}} + \dfrac{1}{C_{\text {it}}} + \dots \right)$. We have accounted for 20 equidistant trap sites inside the oxide for 0.5 nm with $N_{\text {it}}$ = $5 \times 10^{12}$ $\text {cm}^{-2}$ and a deep acceptor trap level ($E_{\text {t}}$) of 0.6 eV relative to the channel (Si) CBM^53^. The carrier capture cross-sections (SIGN and SIGP) are set to $1 \times 10^{-15}$ $\text {cm}^{2}$ each for charge state exchange. Time-dependent charge trapping and detrapping is a function of the $V_G$ sweep rates as calculated from^38^:

5 $$\begin{aligned} \text {SR} =\frac{V_{\text {Gmax}} - V_{\text {Gmin}}}{t_{\text {sw}} \times V_{\text {G,step}}} \end{aligned}$$

where $V_{\text {Gmax}}$ and $V_{\text {Gmin}}$ are the final and initial $V_G$ respectively, $V_{\text {G,step}}$ is the step voltage, and $t_{\text {sw}}$ is the time span for one forward/reverse sweep. Inspired by these Fig. 9a shows the HW in the $I_{\text {D}}(V_{\text {G}})$ forward/reverse sweep at a SR= 0.01 V/s. We perform these simulations by taking into consideration T47D diseased cell owing to it’s superior electrostatic performance so far. The magnified view shows the exact method of HW extraction considering a current criterion ($I_{crit}$ = $10^{-9} A$). Traps contribute to HW only if it captures ($c_t$) a carrier at $V_{\text {Gmax}}$; faster than $t_{\text {sw}}$ but is too slow to emit ($e_t$) before reaching $V_{\text {Gmin}}$; slower than $t_{\text {sw}}$^23^. The forward/reverse sweep results in a triangular waveform over time as shown in Fig. 9b. The Si-SiO$_2$ interface traps are shown in the energy band diagram along the vertical cutline of the top gate (TG) and back gate (BG) stack in Fig. 9c. These acceptor traps (-/0) which are negatively charged when occupied with electrons and neutral when empty. Defects exchange charge with the Si CBM via elastic tunneling^22^. Traps are populated with electrons due to the positive change in the flat-band voltage ($V_G$ > 0 V) as the Fermi level ($E_{\text {F}}$) moves close to the silicon CBM^49,54^. The phenomenon of charge trapping and detrapping at Si-SiO$_2$ interface is schematically shown in Fig. 9d. The pronounced band bending along with the change in the channel surface potential during the up sweep populates interface traps with the electrons from the channel CBM. However, during the down sweep some electrons remain trapped while the others depopulate depending on the trap time constants, which shifts the threshold voltage in the $I_{\text {D}}(V_{\text {G}})$ giving rise to HW. Having established this phenomenon in the simulation framework, we investigate HW behavior by comparing the T47D diseased cell with the MCF-10A healthy cell.

**Figure 10:**
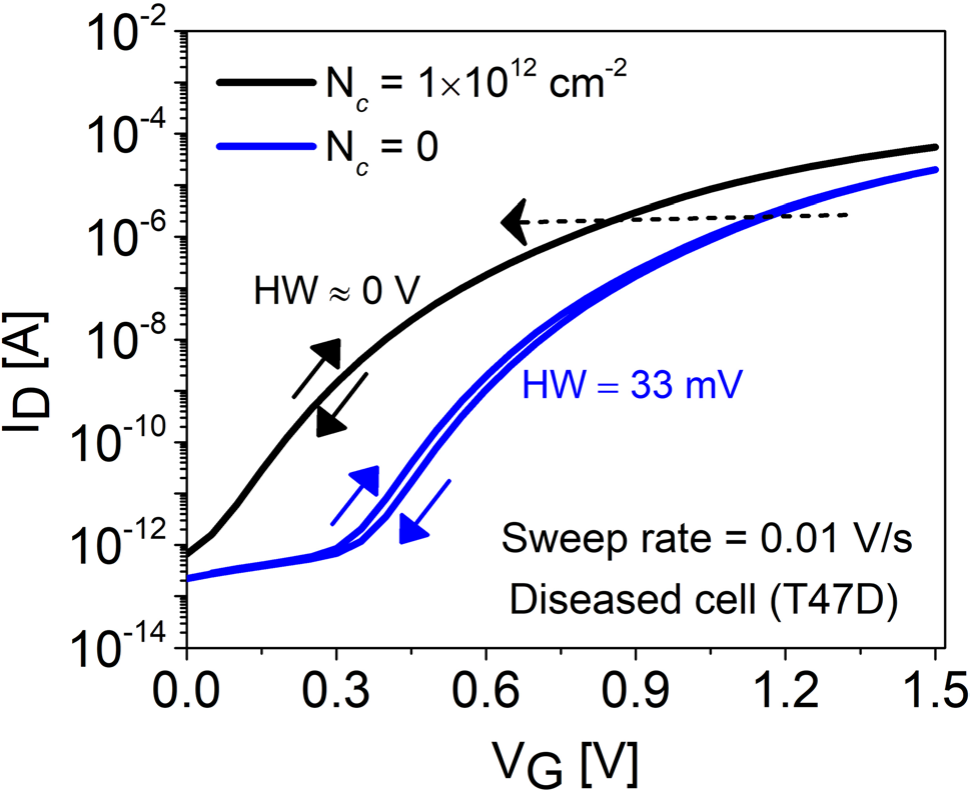
$I_{\text {D}}(V_{\text {G}})$ profile for fixed positive charge associated with T47D cancer cell (black curve) showing HW $\approx$ 0 V while the sensor when operated in the presence of neutral cancer cell T47D and transient (switching) traps result in HW = 33 mV (blue curve) clearly showing that transient traps are suitable for modeling HW in FETs.

Furthermore, we investigated the theoretical origin of hysteresis by simulating forward/reverse sweeping (1) in the presence of diseased cancer cell (T47D) with fixed charges of $N_{\text {c}}$ = +$1 \times 10^{12}$ $\text {cm}^{-2}$ and $N_{\text {it}}$ = 0 , (2) in the presence of neutral biomolecule ($N_{\text {c}}$ = 0) with $N_{\text {it}}$ = $1 \times 10^{12}$ $\text {cm}^{-2}$. As shown from Fig. 10, positively charged T47D cell do not result in observable HW as compared to neutral T47D with switching traps at the Si-SiO$_2$ interface which results in HW $\approx$ 33 mV. This typically occurs because $e_t$ associated with the fixed charges are bias independent at smaller gate voltages unlike the switching traps as explored experimentally^55^ which means that these charges, once captured during the forward sweep, are unable to discharge during the reverse sweep resulting in a constant threshold voltage shift and independent of SR.

In practical biosensing, operation in electrolytes (e.g., PBS) with a functionalized surface (antibodies/aptamers) introduces an electric double layer (EDL) and biolayer capacitances in series with the gate stack, together with fixed and mobile charges at the oxide–electrolyte interface^56,57^. In a compact view, these contributions effectively modify the net capacitance,

$$\left( \frac{1}{C_{\text {net}}}\right) =\left( \frac{1}{C_{\text {ox}}}+\frac{1}{C_{\text {ch}}}+\frac{1}{C_{\text {it}}}+\frac{1}{C_{\text {bio}}}+\frac{1}{C_{\text {EDL}}}+\cdots \right) ,$$

and reshape the surface-potential coupling $\psi _s(V_G)$. Debye charged biomolecules depending on the Debye length $\lambda _D$, which shifts $V_\text {th}$ and can alter the apparent hysteresis width (HW) through a changed occupation of switching traps. Furthermore, mobile ions may introduce additional slow interfacial charge components, effectively adding time constants that can blur or superpose with trap-induced dispersive dynamics. The present work excludes explicit EDL/biolayer transport and focuses on intrinsic reliability (trap-induced hysteresis) in a solid-state framework. TCAD realizations of liquid operation typically solve Poisson–Boltzmann by activating the Gouy–Chapman model^58^ in the electrolyte region and require calibration of ionic properties, reference electrode boundary conditions, and biolayer charge/capacitance. The present reliability methodology is, however, directly extendable: in future work we will incorporate $C_{\text {bio}}$, $C_{\text {EDL}}$, and screened charged biomolecules to co-model sensitivity, hysteresis, and stability under liquid operation.

**Figure 11:**
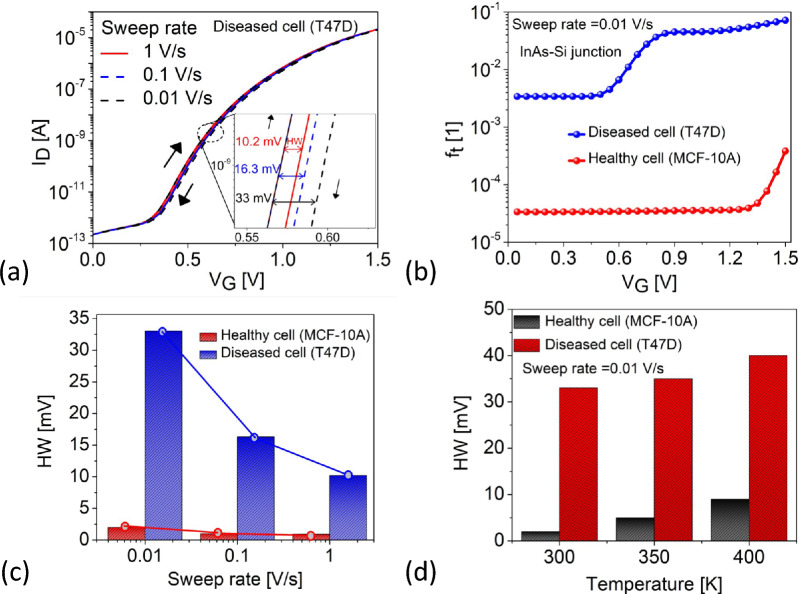
(**a**) $I_{\text {D}}(V_{\text {G}})$ profile with different SR influencing HW for T47D diseased cell (**b**) acceptor trap occupation probability (f$_t$) as a function of gate voltage (V$_G$) for diseased and healthy cell during a forward sweep at room temperature. (**c**) HW as a function of SR and (**d**) temperature for diseased and healthy cells for $N_{\text {c}}$ = 0.

**Figure 12:**
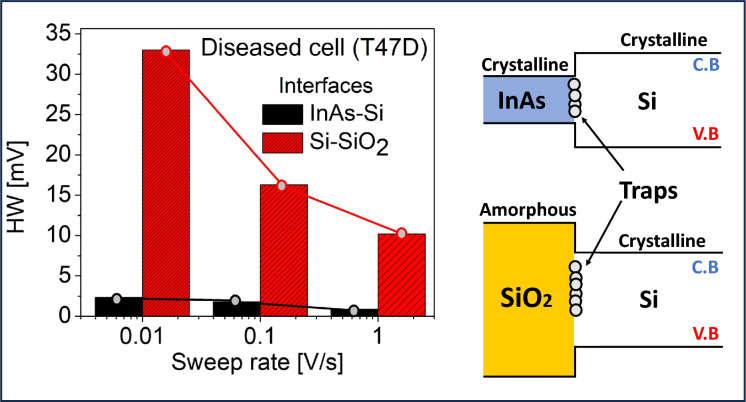
HW as a function of SR from traps at InAs-Si and Si-SiO$_2$ interfaces for T47D diseased cell with the following schematic of energy bands highlighting the impact of material properties on HW. The faster time constants distribution of the crystalline-crystalline interface results in smaller hysteresis when compared to the amorphous-crystalline interface for $N_{\text {c}}$ = 0.

**Figure 13:**
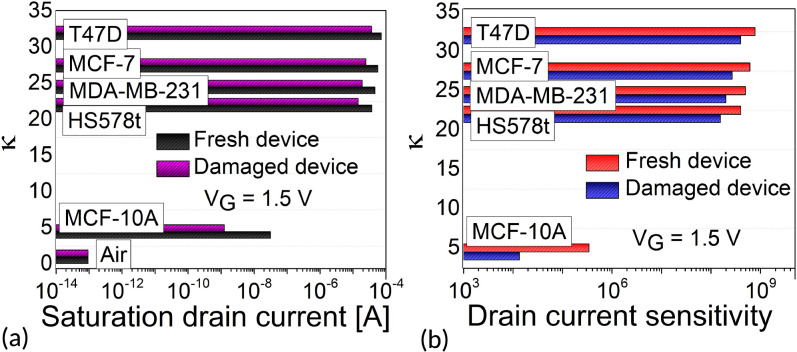
(**a**) Saturation drain current variation. (**b**) Drain current sensitivity with the cancer cells dielectric constant ($\kappa$) for damaged and fresh devices at fixed $V_G$ for $N_{\text {c}}$ = 0.

**Figure 14:**
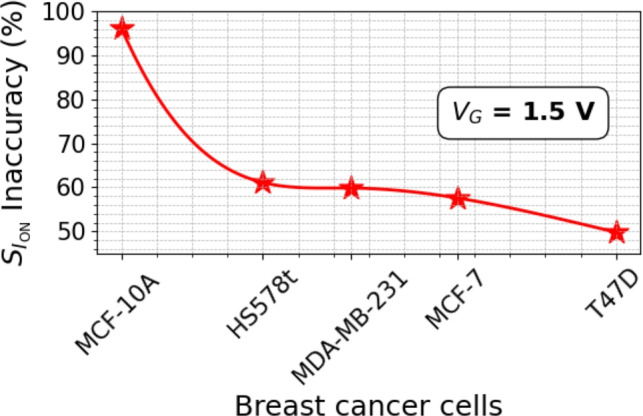
Percentage inaccuracy of drain current sensitivities for damaged and fresh biosensors at room temperature. The detection inaccuracy highlights the crucial need for considering reliability measurements before tape out.

Figure 11a shows HW dependence on SR in the $I_{\text {D}}(V_{\text {G}})$ profile. HW is 10.2 mV for a fast SR (1 V/s) and 32.8 mV for a slower SR (0.01 V/s). Slower SR allow for charge transitions at longer timescales with the Si CBM, increasing the HW. In contrast, faster SR (1 V/s) cause rapid band bending without significant HW. Since, HW is a function of $f_t$, Fig. 11b shows the variation of the acceptor $f_t$ versus $V_G$ at the source-channel tunneling junction. For diseased cell, T47D filled cavity, a single trap starts occupying from $V_G$ $\approx$ 0.4 V whereas healthy cell in the cavity causes minimal charge exchange which is evident in the red curve where the trap occupation only begins at $V_G$ $\approx$ 1.3 V. The occupation probability of a trap is determined by the Fermi-Dirac statistics and the capture-emission time constant dynamics^20,22^:

6 $$\begin{aligned} f_t = \left( \frac{t_{\text {sw}}}{\tau } \right) |\Phi (x)|^2 \Delta d \end{aligned}$$

Here, $\tau$ represents the time constants of the defects (inverse of the transition rates, *k*), $\Phi (x)$ is the wavefunction of the charge states, and $\Delta d$ is the trap distance within the oxide. For cancer cells with higher $\kappa$ values like T47D, $\lambda _W$ reduces, and the vertical electric field aids improving the tunneling rate, meaning greater carrier injection from source to channel. This enhances the probability of trap occupation at the source-channel junction, increasing HW. T47D ($\kappa = 32$) exhibits greater $f_t$ than MCF-10A ($\kappa = 4.5$), leading to increased HW for T47D, as shown across SR in Fig. 11c and Table 5. This demonstrates that fast SR reduce HW, improving device stability. Additionally, HW dependence on temperature is shown in Fig. 11d at a 0.01 V/s SR, where HW increases at higher temperatures (T = 400 K), explained by the time constants^54^:

7 $$\begin{aligned} \tau \propto \exp \left( \frac{E_a}{k_BT}\right) \end{aligned}$$

Here, $E_a$ is the activation energy. Therefore at higher temperatures, the time constants reduces and the thermal velocity increases ($v_{\text {th}} \propto \sqrt{T}$ ) increasing $f_t$ in equation (6), thereby increasing HW. This is summarized under Table 6. As a side note, $\kappa$ of the cells might vary for different temperatures, however, for simplicity, we consider uniform $\kappa$ values.

Furthermore, we also compared the impact of interface trap dynamics at the InAs-Si tunneling hetero junction (owing to their $\approx$ 11 $\%$ lattice mismatch) and the Si-SiO$_2$ interface in Fig. 12. Based on^25^, we modeled the InAs-Si interface traps with a uniform distribution along the $E_g$ and $N_{\text {it}}$ = $1 \times 10^{13}$ $\text {cm}^{-2}$. The crystalline-crystalline interface at InAs-Si results in shorter time constants as compared to the longer time constants associated with the amorphous-crystalline interface at Si-SiO$_2$^19,54^. Therefore, the observed HW for InAs-Si is smaller compared to the Si-SiO$_2$ interface across wide sweep rates implying their $V_G$ insensitivity therefore resulting in less electrostatic degradation.

As a side note, beyond transient hysteresis and trap-assisted degradation, long-term reliability concerns such as bias temperature instability (BTI), hot-carrier effects (HCEs), and device aging can further impact the stability of TFET-based biosensors under repeated operation. These effects promote defect generation and mobility degradation near the tunneling junction and oxide interfaces, progressively altering sensor subthreshold behavior and sensitivity^26,59–62^.

**Table 5:** Impact of SR on HW for healthy and diseased cells.

SR [V/s]	HW (Healthy cell) [mV]	HW (Diseased cell) [mV]
0.01	2.0	32.8
0.1	1.0	16.3
1	0.9	10.2

**Table 6:** Impact of temperature on HW for healthy and diseased cells for fixed SR.

Temperature [K]	HW (Healthy cell) [mV]	HW (Diseased cell) [mV]
300	2.0	32.8
350	5.0	35.1
400	9.0	40.2

**Table 7:** Drain current sensitivity at fixed $V_G$ for fresh and damaged devices.

Cancer cells	fresh device	damaged device
MCF-10A $(\kappa =4.5)$	$3.42 \times 10^{5}$	$1.36 \times 10^{4}$
HS578t $(\kappa =22)$	$4.01 \times 10^{8}$	$1.56 \times 10^{8}$
MDA-MB-231 $(\kappa =24.5)$	$4.98 \times 10^{8}$	$2.0 \times 10^{8}$
MCF-7 $(\kappa =27.5)$	$6.13 \times 10^{8}$	$2.6 \times 10^{8}$
T47D $(\kappa =32)$	$7.77 \times 10^{8}$	$3.9 \times 10^{8}$

**Table 8:** Performance comparison of H-OE-DG-TFET biosensor with existing breast cancer sensors detecting T47D cancer cell at 300 K.

Sl no.	FET-based biosensors	${\boldsymbol{S}}_{I_{\text {ON}}}$	HW [mV]
1	^63^, peak $V_G$ = 0.45 V	$1.1 \times 10^{7}$	-
2	^14^, peak $V_G$ = 0.60 V	$2.88 \times 10^{9}$	-
3	^64^, peak $V_G$ = 0.70 V	$3.5 \times 10^{9}$	-
4	^65^, fixed $V_G$ = 1.5 V	$7.82 \times 10^{10}$	-
5	^66^, fixed $V_G$ = 3.0 V	$2.0 \times 10^{4}$	-
6	^46^, fixed $V_G$ = 1.4 V	$2.3 \times 10^{9}$	-
7	^67^, peak $V_G$ = 0.1 V, $V_D$ = 0.8 V	$9 \times 10^{9}$	–
8	^67^, peak $V_G$ = 0.1 V, $V_D$ = 1.2 V	$8 \times 10^{10}$	–
9	^68^, fixed $V_G$ = -1.25 V	$2.3 \times 10^{8}$	–
10	Our (fresh) sensor, peak $V_G$ = 1.05 V	$1.11 \times 10^{9}$	–
11	Our (fresh) sensor, fixed $V_G$ = 1.5 V	$7.77 \times 10^{8}$	–
12	Our (damaged) sensor, fixed $V_G$ = 1.5 V	$3.9 \times 10^{8}$	10.2 (SR = 1 V/s)

### Sensitivity for a damaged biosensor

Traps at the semiconductor/oxide interface can damage device electrostatics, as discussed in the previous sub-section. These traps introduce recombination centers within the electronic band gap of the channel, degrading $\mu$ and $I_{\text {ON}}$, which in turn reduces $S_{I_{\text {ON}}}$. In this section, we study these effects on biosensing. Since we implement a fixed $N_{it}$ in the simulation framework, changes in SS have negligible impact. Figure 13a shows the degradation of $I_{\text {ON}}$ in the damaged device compared to the fresh device, with observable degradation across different cancer cells linked to the cavity which is mainly attributed to the traps with varying time constants at the Si-SiO$_2$ interface reducing the net available electrons in the channel when compared to a trap-free device. The following $S_{I_{\text {ON}}}$ is calculated and shown in Fig. 13b and Table 7. $S_{I_{\text {ON}}}$ degrades for damaged devices. Based on our calculations, $S_{I_{\text {ON}}}$ inaccuracy percentage from the following relative ratio:

8 $$\begin{aligned} \text {S}_{I_{\text {ON}}}~\text {inaccuracy}~(\%) = \left| \frac{S_{I_{\text {ON}}}^{\text {damaged}} - S_{I_{\text {ON}}}^{\text {fresh}}}{S_{I_{\text {ON}}}^{\text {fresh}}} \right| \times 100 \end{aligned}$$

where $S_{I_{\text {ON}}}^{\text {damaged}}$ and $S_{I_{\text {ON}}}^{\text {fresh}}$ represents the drain current sensitivities for damaged and fresh biosensors and shown in Fig. 14. The detection inaccuracy improves for diseased cells (towards T47D cancer cell), however, the biosensor device nearly fails to sense healthy cells (MCF-10A) with $\approx$ 97% inaccuracy. This can be detrimental in real scenario emphasizing the critical role of charge trapping in the reliability and stability of FET-based sensors. This highlights the need to consider both reliability and sensitivity in biosensor evaluation. Table 8 compares the simulated biosensor with the literature for the detection of T47D cancer cell at room temperature, the drain current sensitivity ($S_{I_{\text {ON}}}$) performances, and the hysteresis width (HW). Unlike previous studies which focused solely on sensitivity, this work addresses both reliability and long-term stability, making it a compact bio sensing study.

**Figure 15:**
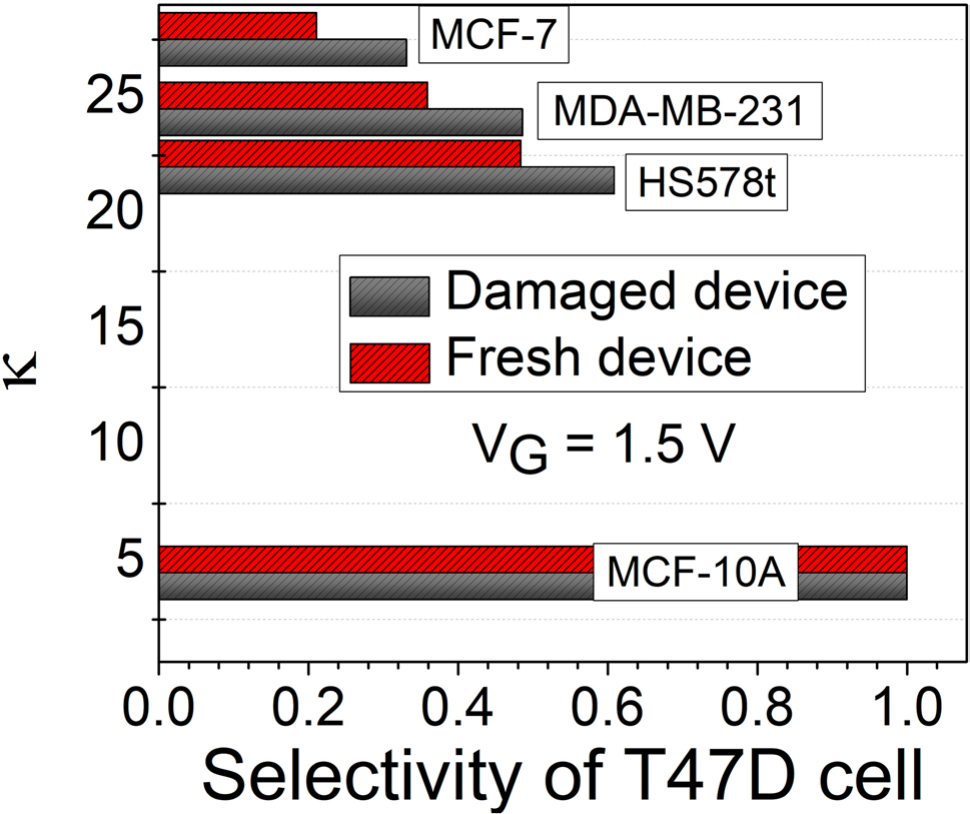
Selectivity of T47D target cancer cell for fresh and damaged devices in the presence of healthy cell (MCF-10A) and other cancer cells (HS578t, MDA-MB-231 and MCF-7) at fixed $V_G$ = 1.5 V for $N_{\text {c}}$ = 0.

### Selectivity for a damaged biosensor

Selectivity plays a crucial role in benchmarking a biosensor to sense a target biomolecule effectively within an environment of other biomolecules. Figure 15 shows the selectivity of the T47D cancer cell for the damaged and fresh sensor device. The sensor selectivity is calculated using the relative ratio considering their saturation drain current ($I_{\text {ON}}$) at $V_G$ =  1.5 V^69^:

9 $$\begin{aligned} \text {Selectivity} =\frac{I_{\text {ON}(\kappa =32)} - I_{\text {ON}(\kappa =4.5, 22, 24.5 { \& } 27.5)}}{I_{\text {ON}(\kappa =32)}} \end{aligned}$$

The calculated selectivity values reveal a notable difference between fresh and damaged biosensor devices, where the selectivity increases with the change of the dielectric constants of the cancer cells relative to the T47D target cancer cell. For both fresh and damaged devices, the selectivity is maximum ($\approx$ 0.99) for MCF-10A healthy cells ($\kappa$ = 4.5). This indicates a moderate discrimination capacity between the T47D cell ($\kappa = 32$) and other breast cell lines. However, selectivity decreases for MCF-7 cancer cells ($\kappa$ = 27.5) by 79$\%$ for fresh devices and only 67$\%$ for damaged devices compared to healthy cells. This counterintuitive increase in selectivity for damaged devices can be explained by modifying the interfacial charge dynamics and dielectric screening. During degradation, the formation of interface traps or fixed charges alters the local electric field distribution within the sensing channel. However, it is important to note that this higher selectivity does not necessarily indicate better biosensing performance. The overall saturation drain current of the damaged device typically degrade, meaning that although the relative difference between cells increases, the absolute signal levels decrease. In practical terms, the sensor becomes more selective but less sensitive, reflecting a trade-off between device degradation and dielectric contrast amplification.

## Conclusion

This work presents a simulation-based investigation of a heterostructure oxide-engineered double-gated TFET biosensor for breast cancer detection, emphasizing its modeled sensitivity, reliability, and stability characteristics. Our analysis reveals that higher dielectric constants of diseased cells lead to enhanced electrostatic coupling and increased drain current sensitivity, making the device highly suitable for distinguishing cancerous from healthy cells. Furthermore, the study of hysteresis effects and their dependence on SR and temperature provides critical insights into the reliability of the biosensor under varying operational conditions, such as faster SR and lower temperatures, which reduce HW. This work introduces a novel sensitivity inaccuracy metric that quantifies nearly 97% loss of sensitivity in damaged biosensors. By integrating performance metrics with reliability assessments, this research provides predictive guidance for the design of reliable biosensors for early breast cancer detection, while recognizing that experimental validation is required to confirm practical applicability. The simulations clarify that fixed positive charges from cancer cells shift transfer curves but do not cause hysteresis, while switching traps at the Si–SiO_2_ interface do; the resulting HW scales strongly with sweep rate (10.2 mV at 1 V/s versus 32.8 mV at 0.01 V/s) and with temperature (up to $\sim$40.2 mV at 400 K), and is consistently larger than at the InAs–Si interface. Finally, we report how device degradation can alter selectivity among cell lines, underscoring the need to co-report sensitivity, hysteresis, and the proposed inaccuracy metric in future TFET biosensing studies. All results originate from calibrated TCAD simulations, and no measurements on fabricated H-OE-DG-TFET biosensors or liquid-phase breast-cell assays are included. Operation in realistic electrolyte environments with functionalized gate stacks is not modeled self-consistently here; effects such as electric double-layer formation, biolayer capacitances, and interfacial charge dynamics under liquid conditions are only discussed conceptually. In addition, the trends in hysteresis and sensitivity rely on assumed trap energy distributions, and biomolecule dielectric constants from literature, which may vary in fabricated devices. A key takeaway is that HW can be considered as a sensing metric that offers predictive insight to support future experimental validation.

## Data Availability

All data generated or analyzed during this study are included in this manuscript.
